# Effect of blended fertilizer rates and planting density on yield and yield components of Irish potato (*Solanum tuberosum* L.) at Gombora condition, Hadiya zone, Southern Ethiopia

**DOI:** 10.1016/j.heliyon.2023.e17450

**Published:** 2023-07-01

**Authors:** Temesgen Tadesse, Getachew Mulugeta

**Affiliations:** a Wachemo University, College of Agriculture, Department of Horticultural Sciences, P.O.B. box 667, Ethiopia; b Wachemo University, College of Agriculture, Department of Natural Resource Management, Ethiopia

**Keywords:** Blended NPS fertilizer, Crop management, Intra-row spacing, Marginal rate of return, Planting density, Yield

## Abstract

Potato is an important food and cash crop and it has high yielding potential in many parts of Ethiopia; however the yield of the crop is often constrained due to low and imbalanced rates of inorganic fertilizers and inappropriate spacing. The field experiment was conducted to determine the effect of five rates of blended fertilizer (0, 100, 150, 200, and 250) kg NPS ha^−1^ and intra-row planting spacing of 20 cm, 30 cm and 40 cm and laid out by randomized complete block design with three replication in a factorial arrangement. The analysis of variance revealed that, marketable tuber yield, total tuber yield, stem number per hill, total fresh mass, underground fresh and dry mass were significantly (P < 0.05) influenced by the interaction of Nitrogen, Phosphorous and Sulfur (NPS blended fertilizer) and intra-row spacing. The highest plant height (96.60 cm), highest marketable tuber yield (34.29 tha^-1^), highest total tuber yield (38.36 t ha^−1^) and highest total fresh biomass (1274.2 g plant^−1^) were recorded from NPS rate of 250 kg NPS ha^−1^ and intra-row spacing of 20 cm while the lowest recorded from control treatment in wider intra-row spacing (40 cm). Therefore, the application of 250 kg NPS ha^−1^ with the intra-row spacing of 20 cm can give an optimum tuber yield and it could be recommended for the production of potato in the study area.

## 1 Introduction

Potato (*Solanum tuberosum* L.) is the world's fourth most important food crop after maize, rice and wheat. It is an important source of carbohydrate, protein, vitamins B and C and also minerals. It provides a source of income and is important in food and nutritional security [1]. It is an herbaceous annual crop cultivated for a tuber in which edible food materials are stored. The crop is native to South America and has been introduced to Ethiopia since 1859 by a German Botanist called Schimper [2]. It has been identified as a cheap source of human diet, since it produces more food value per unit time, land and water than any other major crops [3].

It is an important food and cash crop in Eastern and Central Africa. It plays a major role in national food security, nutrition, poverty alleviation, income generation, and increased employment in production, processing and marketing sub-sectors [4]. Among root crops, potato ranks first in volume produced and consumed, followed by cassava, sweet potato, and yam [5]. In Ethiopia, its winter season production area has reached about 66, 926 ha, total production of 921,403.2 ton cultivated by over 1.2 million households [6]. On the other hand, the national productivity of this crop in the country is very low (13.8 t ha^−1^) compared to the world's average yield of 19 t ha^−1^ and from the experimental yields of 40.2 t ha^−1^ [7]. Potato is one of the high yielding crops in many parts of Ethiopia. However; its production is limited due low soil fertility and disorganized management practices. Thus the low soil fertility problem is due to its nutrient depletion because of continuous cultivation without adequate replenishment of the mined nutrients and this can limit the crop yield [8]. And also nutrients such as, Nitrogen, Phosphorous and Sulfur can be absorbed in large quantity during the whole growing period of the crop [4].

Nitrogen is an integral component of many compounds such as chlorophyll, nucleotides, alkaloids, enzymes, hormones and vitamins and these are essential for plant growth processes [9]. It is a nutrient that can frequently limits yield and quality of potato crop [8]. Insufficient available N leads to reduce growth and reduce light interception [10], limits yields [6] and early crop senescence [11]. Excessive available N can result in reduced yields [12] especially, in late-maturing varieties [13]; delayed tuber set and reduced tuber dry matter content [8]. Excess amount of N extraordinarily increases also shoot development, which in turn decreases photosynthesis/respiration ratio, then less assimilate is transported to tubers [14]. The main role of N is in swift development of shoot and allows the plant to quickly complete its canopy and exploit the growth period as much as possible. N increases photosynthetic/respiration ratio through increasing mature leaf number and hence, improves yield [3]. [15], reported that N increases shoot dry weight. Leaf to stem ratio was found also to be increased by nitrogen [10]. Therefore, fertilization with N changes the chemical composition of plants [12] by increasing N content in tubers.

This crop has high demand for phosphorus (P) as compared to other crops [16]. P is important for vine growth, tuber formation, and tuber bulking and tuber starch synthesis [17,18]. The crop has a low P uptake efficiency due to its shallow root system. Majority of roots can be found in the top 30 cm of the soil [14]. Differences in the rooting systems and utilization ability of genotypes influence optimum rates to be applied. To get the most benefit from fertilizer application, placement and rate of application is critical. The risk of P losses in surface runoff is higher with very high P concentrations in soil [17]. Application of appropriate rates will also minimize risk of contamination of water sources and reduce production costs [18].

Sulfur is one of 16 essential nutrient elements and fourth major nutrient after NPK, required by plants for proper growth and yield as it is known to take part in many reactions in all living cells [9]. The content of sulfur in potato tubers is on average between 0.7 and 2.0 g kg^−1^ and its uptake ranges from 18 to 40 kg ha^−1^ [19]. It increases the resistance of this cultivar of potato to environmental stress and plays an important role in protecting the plants from pests and diseases. Sulfur deficient plants had poor utilization of nitrogen, phosphorus and potash [15]. It can also enhance starch synthesis in tubers and it is a component of proteins and many enzymes. This nutrient plays an essential role in chlorophyll formation and therefore helps to give plants their green color [13]. It is known to take part in many reactions in all living cells [20]. It is a key component of balanced nutrition required for the production of potato where intensive cropping and use of high grade fertilizers resulted in depletion of soil sulfur [21].

The soil test through Ethio-SIS related that, Ethiopian soils are deficient in other micronutrients like Sulfur and others. This is attributed to high soil erosion, removal of nutrients by crops and continuous cropping with no replenishment of nutrients and inadequate and unbalanced use of chemical fertilizers [22]. Soil fertility depletion owing to high rates of erosion is considered to be the fundamental biophysical root cause for declining per capita food production in Africa, including Ethiopia in the fields of smallholders [23].

Additionally, increment in the number of plant population per unit area due to plant spacing has direct effect for the increment of potato tuber yield and also the pattern in which the given amount of seed or plant density is arranged in the field of planting [8]. Effect of increasing plant population per hectare on potato has revealed high increment on the yield of the crop as reported by Ref. [11]. And then, providing adequate plant density and amending low soil fertility by applying inorganic fertilizer should be considered to address problems among potato production. Therefore, its production and productivity can be increased by providing proper management practices.

It is known that potato crop requires large amount of essential mineral elements for its better growth and yield because it is high feeder of nutrients. Even if the study area is suitable and potential for this vegetable production, there is still low productivity due to inadequate rate of inorganic fertilizers, inappropriate row spacing and lack of improved varieties. And also, most of the farmers are cultivating this crop by applying low rates of inorganic fertilizer which is below 100 kg NPS ha^−1^ and inadequate plant spacing which is far closer or wider than the nationally recommended intra-row spacing of 30 cm [11]. This has contributed for low productivity of the crop. And also there is no research conducted regarding on optimum rate of Nitrogen Phosphorous and Sulfur (NPS) blended fertilizer and appropriate intra-row spacing specific to the study area.

Consequently, this study was conducted to evaluate effects of Nitrogen, Phosphorous and Sulfur (NPS) blended fertilizer and intra-row spacing on yield and yield components of potato at Gombora district since the area is potential for potato production in Hadiya Zone, Ethiopia.

## 2 Materials and methods

### 2.1 Description of study area

The field experiment was conducted at Gombora district in Southern Ethiopia during 2019 summer cropping season. Gombora district is chosen by considering its potential for potato production. The experimental site is located 8 km west of the town of Hossana at Hadiya Zone. Geographically the site is located on 7014ꞌ to 7° 45ꞌ North latitude and 370 5ꞌ to 37° 50ꞌ East Longitude at an altitude of 2350 m above sea level (m.a.s.l). This represents a high altitude and high rain fall environment. The climatic condition of the area is moist humid and sub humid moisture condition and suitable for potato production. The rainy season extends from January to March, where the maximum rainfall is received in the months of February and March. And regarding the climatic condition, the area receives average annual rainfall of in between 1000 mm and 1400 mm and the average minimum and maximum air temperature is 12^o^_c_ to 16^o^_c_ respectively and characterized as humid agro-ecological zones.

### 2.2 Treatments and experimental design

Belete variety of potato was used as the experimental material. The tuber seed of this variety was collected from Holleta Agricultural Research Center, Ethiopia. It could grow well with in the altitudes range 1600–2800 m.a.s.l. Blended fertilizer product was used as source of Nitrogen, Phosphorous and Sulfur (NPS) fertilizers. The experiment comprised five rates of NPS blended fertilizer; such as control or (0), 100, 150, 200, 250) NPS kg ha^−1^ and three intra-row spacing of 20 cm, 30 cm and 40 cm. According the report of [24] it is known that the content of (N = 19, P_2_O_5_ = 38 and S = 7) kg in 100 kg of NPS blended fertilizer. A randomized complete block design with three replications was used. Each plot have consisted 66,667, 44,444 and 33,333 plants per hectare with respective intra-row spacing of 20 cm, 30 cm and 40 cm. The size of each plot is 3.75 m * 3.6 m = 13.5 m^2^ by providing 1 m space between block and 0.5 m between plots. Weeding and other management practices were carried out manually and frequently to manage the field. The NPS blended fertilizer was applied at once during planting while urea fertilizer was applied in split application with the same rate of 150 kg urea per hectare. One third of urea fertilizer was applied during planting and the rest two-third after 45 days of planting.

### 2.3 Soil sampling and analysis

A composite soil sample of 0.5 kg from the depth of 0–30 cm was collected from the experimental site before planting from 10 representative spots. The collected soil samples were air-dried, crashed and sieved through 2 mm mesh to perform the analysis soil Physio-chemical characteristics. And the soil organic matter was determined by following [25] method. Available P was determined by Ref. [26] method. The soil had medium OC in accordance with [27]. [28], classified organic matter content of 3–5% as high and more than 5% as very high. But, soil with OC 1.24% which is rated low according to Ref. [29]. Thus the experimental site of available OM content was rated as high (2.14%). According to Ref. [26], P content of <3 is very low, 4 to 7 is low, 8 to 11 is medium, and >11 is high. Total N of the soil (0.11%), is low; as rated by Ref. [28]. The pH of the soil was 6.2, which is within the range of 5.5–6.8 suitable for potato production [29]. Soil pH was determined in 1:2.5 soil: water ratio using a glass electrode attached to a digital pH meter.

### 2.4 Data collection

#### 2.4.1 Days to emergence

The number of days from planting to 50% emergence was used as days to emergence for statistical analysis. Days to 50% flowering: This was recorded by counting the number of days from planting to when 50% of plants in each plot were flowered. Days to 90% maturity: was recorded when 90% of the plants in each plot were ready for harvest the senescence of leaves and haulms has attained and used for the analysis. Plant height (cm) of five randomly selected plants from the middle rows were measured from the ground surface to the tip of the main stem at physiological maturity stage. Average stems number per hill: Number of stems raised from the ground from randomly selected five plants was counted when 50% of the plants in each plot attained flowering stage and mean number of stem per hill was used for statistical analysis. Leaf area (cm^2^): The individual leaf area from five randomly selected potato plants was calculated by using portable leaf area meter model (LI-3000C, panomex Inc.). Total fresh mass (g plant^−1^): It was measured separately for root (roots, tubers, stolen) and shoot (stem, branch, and leaves) from five sampled plants at physiological maturity when the plants was fully develop and practically cease to grow. Total dry mass (g plant^−1^): Dry weight of shoot and underground parts were taken from randomly selected plants and air-dried for seven days and then moved to oven-dry at 72 °C until a constant mass was achieved. Shoot fresh and dry mass (g plant^−1^): The leaves and stems from randomly selected five plants were measured and taken as the mean shoot fresh weight of a crop. And sampled plants were air dried for seven days and then oven dried at 72 °C for 24 h and the average dry weight was considered for statistical analysis. Underground fresh and dry weight (g plant^−1^): The sample taken for fresh weight measurement was used for dry weight measurement by drying the roots, stolons and the tuber parts for seven days in the sun and moved to an oven drying at 72 °C until a constant weight was reached, then after weighted and averaged as dry shoot mass yield per plant in each plot.

#### 2.4.2 Average tuber number per hill

All plants were harvested and the average was taken by dividing the number of tubers to the number of plants. The average tuber number per hill was used for statistical analysis. Average tuber weight (g/tuber): This was determined by dividing total tuber yield (weight) harvested from randomly sampled five plants to total tubers numbers harvested from five plants. Marketable tuber yield (t ha^−1^): Tubers which are free from diseases, damages, insect pests with mean weights of above 25 g was collected from net plot area. Unmarketable tuber yield (t ha^−1^): Weight of tubers unhealthy, injured by insect pests and the size less than 25 g was recorded from net plots harvested. Total tuber yield (t ha^−1^): The total tuber yield per hectare was recorded by sum of weights of marketable and unmarketable tubers expressed in t ha^−1^ for analysis. Tuber dry matter: Five tubers representing all size categories of the variety was collected and chopped into 1–2 cm small cubes. Dried samples were weighed by using sensitive balance but the dry matter content was determined using the following formula: Dry matter content (DM) % = ((Dry weight/Fresh weight) x 100) according to CIP (2006). Harvest index: was calculated as the ratio of dry mass of tubers to the total biomass dry yield. Firstly, tubers from five randomly selected plants were obtained and dried in the sun for the consecutive five days and placed in the oven dry with 72 °C until constant dry weight was attained and the total dry matter was also performed.

$$Harvest\;Index\;\left(HI\right)=\frac{Tuber\;dry\;matter}{Total\;dry\;matter}$$

### 2.5 Data analysis

The collected data were analyzed using SAS statistical software package updated version 9.3 [30]. And the means which exhibited the significant differences among treatments were separated by using LSD test at 5% level of significance. This was done depending on the ANOVA result that showed the presence of significant difference among the treatments.

## 3 Results and discussion

### 3.1 Days to 50% emergence

The result showed that effect of blended NPS fertilizer application significantly (*P* < 0.05) delayed the time required to attain 50% emergence of potato. Average days to 50% emergence ranged from (19.0) days to (23.7) days. The minimum and maximum number of days to reach days to 50% emergence was recorded for the application of 0 kg NPS ha^−1^ and 250 kg NPS ha^−1^ fertilizer rates respectively (Table 1). The result of present study was supported by (Kinde and Asfaw 2016) who reported that increased application of blended fertilizer delayed the time to attain 50% emergence by 6.0 days and this might be due to the role of increased N and P fertilizer contents.

**Table 1:** Days to 50% emergence, days to 50% flowering days to 90% maturity, plant height and leaf area of potato crop was affected by blended NPS fertilizer rates and intra-row spacing at Gombora condition during 2019 summer cropping season, Hadiya Zone, Ethiopia.

Treatments	Days to 50% emergence	Days to 50% flowering	Days to 90% maturity	Plant height cm	Leaf area (cm)
NPS (kg ha^−1^)					
0	19.00^c^	60.77^c^	103.86^e^	69.62^e^	20.01^e^
100	19.78^d^	64.22^b^	108.53^c^	81.67^d^	21.76^d^
150	21.11^b^	64.22^b^	105.86^d^	87.87^c^	22.71^c^
200	21.56^b^	65.66^b^	114.08^b^	92.22^b^	24.15^b^
250	23.67^a^	70.22^a^	117.97^a^	96.60^a^	25.48^a^
LSD (0.05)	0.62	1.90	1.26	2.95	0.71
Spacing (cm)					
20	21.05^c^	64.27^c^	111.70	101.79^a^	20.67^c^
30	21.77^b^	66.66^b^	112.92^b^	86.28^b^	23.47^b^
40	22.55^a^	69.50^a^	114.53^a^	73.46^c^	26.03^a^
LSD (0.05)	0.44	1.34	0.89	3.08	0.51
CV (%)	4.36	5.05	8.48	13.60	10.28
Mean	21.31	65.69	111.18	86.19	23.03

And also the minimum and maximum days to 50% emergence has ranged from 21.05 days to 22.55 days by widening the intra-row spacing from 20 cm to 40 cm (Table 1). The prolonged days might be due to lesser competition for resource like water, light and nutrients and poor nutrient use efficiency of the crop because of the wider spacing. In this experiment, earlier plant emergence was obtained in closer intra-row spacing (20 cm) and the delayed time to attain days to 50% emergence was obtained at wider intra-row spacing (40 cm. This result was in line with the findings of [24,31] who reported that increasing intra-row spacing resulted in delayed time required to reach 50% emergence.

### 3.2 Days to 50% flowering

The use of NPS blended fertilizer and intra-row spacing has revealed significant influence (*P* < 0.05) on days to attain 50% flowering of potato crop. Mean days to 50% flowering ranged from 60.77 days to 70.22 days. And the faster and delayed days to 50% flowering was recorded from 0 kg NPS ha^−1^ and 250 kg NPS ha^−1^ respectively (Table 1). This delayance might be due to the role of increased application of nitrogen with blended NPS fertilizer. And which as part of the structure of DNA, RNA, ATP and phospholipids in membranes for potato plant in carbohydrate metabolism and energy transfer systems. N has high influence in delaying the flowering of potato by prolonging its vegetative growth [32]. Optimum rates application of fertilizer might led to a general increment of most metabolic processes; however extremely increase in N fertilizer rate can delay the time to flowering [33]. In the same way, varying intra-row spacing showed significant influence (*P* < 0.05) on days to 50% flowering of potato. Minimum and maximum days to 50% flowering ranged from 64.27 days to 69.50 days (Table 1). The delayed flowering of the crop at wider plant spacing might be due to the space which allows less competition for sun light, water and nutrient and this enabled the crop to maintain physiological activity for a long period, thereby continuing photosynthesis. [34,33], has reported significant differences in days to 50% flowering for potato crop and they reported that, the wider the plant spacing, the delayed to attain 50% flowering.

### 3.3 Days to 90% maturity

In this study, the main effect of NPS blended fertilizer and intra-row spacing has significant influence (*P* < 0.05) on the number of days to achieve 90% maturity. Thus, the mean days to 90% maturity has ranged from (103.86–117.97) days due to the application of different rates of blended fertilizers. Maximum mean days to 90% maturity were recorded from application of 250 kg NPS blended fertilizer ha^−1^ whereas the minimum days were attained from control treatment. This delaying might be due the role of Nitrogen fertilizer in extending the vegetative growth of the crop. And also P attributed this to sustained physiological activities of the plants excessive accumulation of photosynthetic assimilates that lead to continued photosynthesis and growth of the plants [17]. Therefore, the present investigation was supported by the findings of [35] who reported extending maturity of potato was observed with the increased rate of N and P inorganic fertilizers.

Mean maximum of (114.53 days) to 90% maturity of potato were recorded at wider intra-row spacing (40 cm) and minimum of (111.70 days) were recorded from 20 cm intra-row spacing. The result indicated that wider plant spacing allowed lesser competition for sun light, water and nutrient which enhanced potato plant to maintain physiological activity for a long period. This result was supported with the findings of [34,36], who reported that decreasing intra-row spacing resulted in shortening the time required to reach 90% maturity.

### 3.4 Plant height (cm)

Analysis of variance (ANOVA) value reveals that, the use of NPS blended fertilizer and intra-row spacing has significant effect (*P* < 0.05) on days to attain 50% flowering of potato crop. Increased application of NPS fertilizer from 0 to 250 kg NPS ha^−1^ had increased the plant height from (69.62 cm) to (96.60 cm) and that the highest plant height was recorded when 250 kg NPS ha^−1^ and the lowest plant height (69.62 cm) which was recorded when zero fertilizer rate/0 kg NPS ha^−1^ (Table 1). There is significant and linear increase in plant height in response to increasing the rate of NPS blended fertilizer application and this may be attributed to the critical role phosphorus, Nitrogen and Sulfur and which plays in enhancing cell division, growth, and stem elongation to meet the demand for the increased plant height [22,37].

And also widening the intra-row spacing from 20 cm to 40 cm had significantly influenced the plant height at which it was increased from (73.46 cm) to (101.79 cm) respectively. The highest plant height (101.79 cm) was recorded from closer intra-row spacing and the shorter plant height (73.46 cm) was attained from the wider intra-row spacing of 40 cm. The taller plant growth in the narrower might be attributed due to the stiff competition for sun light in closer intra-row spacing. Generally, closer spacing stimulated plants to grow taller with sufficient NPS fertilizer in the soil in order to meet the light demand of the crop. This result has supported by the finding of [31,38], that the highest plant height was recorded from closer or narrower intra-row spacing.

### 3.5 Leaf area (cm^2^)

Blended fertilizer rates and intra-row spacing has significant effect (*P* < 0.05) on the leaf area expansion of potato crop. Thus, the widest leaf area per hill (25.48 cm) was obtained at a fertilizer rate of 250 kg NPS ha^−1^ than the leaf area per hill which was recorded at control or at blended fertilizer application of 0 kg NPS ha^−1^ which is about (20.01 cm) Table 1. At higher rate of NPS blended fertilizer application, plants might observe sufficiently available resource, wider ability to get them and increased photosynthetic efficiency with the help of those essential nutrients from blended inorganic fertilizer that further increased the vegetative growth and ultimately resulted in increased leaf area per hill. Therefore, the increased leaf area expansion rate at higher rates of NPS fertilizer might be due to increased epidermal cell expansion with the sufficiency of nutrients. In consistent to this study [39], confirmed that leaf area per hill increased in the high fertilizer rate and that the wider leaf area per hill was obtained from higher fertilizer rate application.

And also widening plant spacing from 20 cm to 40 cm had increased the leaf area per hill from (20.67 cm–26.03 cm). That the largest leaf area per hill (26.03 cm) was obtained from the wider intra-row spacing of 40 cm and the smaller leaf area per hill (20.67 cm) was recorded at closer intra-row spacing of 20 cm. In similar manner [36,40], stated that the largest leaf area hill^−1^ was obtained in the wider intra-row spacing (30 cm).

Consequently, the increased leaf expansion rate at wider intra-row spacing of 40 cm might be either due to increased epidermal cell expansion due to lesser plant population which allows free space that a given crop can occupy and so that the crop can grow in free manner [[41], [42]].

Whereas, means followed by the same letter(s) are not significantly different at *P* < 0.05. NPS= Nitrogen, Phosphorous and Sulfur blended fertilizer, SD = least significance difference, CV = coefficient of variance.

### 3.6 Average stem number per hill

Application of different rates of NPS blended fertilizer and intra-row spacing has significant effect (*P* < 0.05) on the stem number of potato crop. The increased number of stem per hill (9.66) was obtained with fertilizer rate of 250 kg NPS ha^−1^ in wider intra-row spacing of 40 cm and the lower number of stem per hill (8.66) was recorded in the same rate of blended fertilizer rate of 250 kg NPS ha^−1^ at closer intra-row spacing of 20 cm. However, the least number of stems per hill (4.66) were recorded in the control at closer intra-row spacing or from blended fertilizer rate of 0 kg NPS ha^−1^ at closer intra-row spacing (Table 2). There is an increment on the average stem number per hill of potato crop with an increased application of N, P and S blended fertilizers. This might be due to the fact that stem number could also be determined by nutrient application in addition to the early ontogeny of yield potential and the free space around the crop root [6,16].

**Table 2:** Average stem number per hill of potato was influenced by the interaction effect of NPS blended fertilizer and intra-row spacing at Gombora condition at Hadiya Zone, 2019 cropping season.

Average stem number per hill (count)					
	NPS fertilizer rates (kg ha^−1^)				
Spacing (cm)	0	100	150	200	250
20	4.66h	5.00gh	6.33f	8.66cd	8.66cd
30	5.00gh	6.00f	7.66e	8.33de	9.33bc
40	5.66 fg	6.33f	8.66cd	9.66b	9.66b
LSD (0.5%)	0.85				
CV (%)	8.66				
Mean	7.31				

However [43], stated that stem number is basically determined by the number of eyes present on tubers seed and the physiological age of the tubers during the storage period but in some amount by manipulating the supply of fertilizers could have a considerable effect. This increment in number of stem per hill with the increased application NPS fertilizer with the combined effect of wider intra-row spacing might be due role of N, P and S which found in this fertilizer that could trigger the initiation and energy transfer of a crop and the availability of resources [16,28].

Similar with the results of this study, some researchers reported that stem number per hill was significantly affected by the application of phosphorus fertilizer and intra-row spacing [31,33,36].

Whereas, means followed by the same letter(s) in rows and columns under each parameter are not significantly different at *P* < 0.05 level of significance, NPS= Nitrogen, phosphorous and sulfur blended fertilizer, LSD = Least significance difference, CV = coefficient of variation.

### 3.7 Average tuber number per hill

Analysis of variance shown that the application of blended fertilizer and intra-row spacing has significant effect (*P* < 0.05) on the average tuber number per hill of potato crop. In this study increasing the rate of NPS fertilizer from 0 to 250 kg ha^−1^ increased average tuber number per hill by about (65.83%). The enhanced average tuber number per hill was recorded in response to applying 250 kg NPS ha^−1^. However, the lowest tuber number per hill was obtained in the control treatment of 0 kg NPS ha^−1^ (Table 3). Correspondingly [23], have found that increasing the rate of phosphorus fertilizer significantly increased average tuber number per hill of potato. This P fertilizer could have promoted the growth and photosynthesis rate of the plants and tuber formation.

**Table 3:** Tuber number per hill, Average tuber weight, unmarketable tuber shoot fresh and dry weight and harvest index yield of potato as influenced by the increased application of blended fertilizer and intra-row spacing at Gombora condition, Hadiya Zone, Ethiopia.

Main effect	TNPH	ATW (g/plant)	UMTY (tha^−1^)	SFW(g plant^−1^)	SDW (g plant^−1^)	Harvest index
NPS (kg ha^−1^)						
0	15.98^e^	57.96^e^	3.69^b^	261.13^c^	68.41^c^	0.59^d^
100	19.65^d^	64.43^d^	4.34^a^	349.16^b^	81.47^b^	0.65^c^
150	23.88^c^	73.52^c^	4.11^ab^	362.44^b^	94.96^a^	0.69^ab^
200	34.32^b^	75.34^b^	3.40^b^	370.82^ab^	97.15^a^	0.71^a^
250	46.77^a^	77.45^a^	4.48^a^	385.38^a^	100.97^a^	0.74^a^
LSD (0.05)	1.77	1.25	0.51	17.42	9.86	0.03
Spacing (cm)						
20	34.54^a^	66.18^c^	3.67^a^	331.80^c^	86.93^c^	0.67^c^
30	31.87^b^	72.49^b^	3.10^b^	368.62^b^	94.56^b^	0.69^b^
40	31.04^b^	75.55^a^	2.63^c^	386.51^a^	101.27^a^	0.71^a^
LSD (0.05)	0.82	0.88	0.36	13.00	6.30	0.01
CV (%)	15.24	13.26	17.18	14.85	12.20	13.96
Mean	29.76	70.37	3.68	351.98	90.72	0.68

*Means followed by the same letter are not significantly different at *P* < 0.05 level of significance, LSD = Least significance difference and CV = coefficient of variation, ATW = Average tuber weight, ATNPH = Average tuber number per hill, UMTY= Unmarketable tuber yield, SFW= Shoot fresh weight, SDW= Shoot dry weight and HI= Harvest index.

Marketable tuber yield (tone ha^−1^).

An increment in the average numbers of tubers per hill at closer plant spacing might be due to the higher canopy coverage of the ground with green leaves earlier at earlier growth season and light is intercepted and used for assimilation, and also could be due to the higher competition for growth factors like sun light, water nutrients and air and using them in efficient manner. In similar to the current study, decreased plant population density revealed increased small sized tubers per hill was reported by different scholars [41]. Yet, this the present study finding opposes with that of [5,44] who reported lower tuber numbers per hill at closer plant spacing than the wider plant spacing.

### 3.8 Mean tuber weight (g per plant)

Application of blended fertilizer and intra-row spacing has significant effect (*p* < 0.05) on the mean tuber weight of potato crop. The increased mean weight of tubers 77.45 g plant^−1^ was observed in the treatment that received 250 kg NPS ha^−1^ and the lowest mean weight of tubers (57.96 g per plant) was obtained from the control treatment. Increment on the mean tuber weight with the increased application of blended fertilizer might be due to the role of N, P and S fertilizers in this blended fertilizer and that can trigger tuber enlargement and initiation of cell expansion and production in the crop. Similar to the result of this study [3,6,45], who reported that the heavier average tuber weight were obtained from the increased application of NP fertilizer. This increment in tuber weight can be associated with adequate photosynthetic products translocated to the reproductive structures [[9], [46], [47]].

The effects different plant spacing has significantly influenced (*P* < 0.05) the mean tuber weight as shown in (Table 3). Thus, plants grown in wider spacing shown the larger mean tuber weight while, those grown in narrower intra-row spacing had shown the lower mean tuber weight. And the highest mean tuber weight was recorded at wider intra-row spacing of 40 cm and the lowest mean tuber weight was obtained from closer intra row spacing of 20 cm. This result is also in agreement with [5,48], who reported the highest average tuber weight from the effect of wider intra row spacing.

### 3.8 Unmarketable tuber yield (t ha^−1^)

The unmarketable tubers yield per hectare has significantly influenced (*P* < 0.05) by increasing the rate of NPS blended fertilizer from 0 to 250 kg NPS ha^−1^ and intra-row spacing from 20 cm to 40 cm (Table 3). Higher unmarketable tuber yield was obtained from the plots which are treated with 250 kg NPS ha^−1^ and the lower unmarketable tuber yield was recorded control treatment. This may be due to the role NPS fertilizer which can highly contribute for increased tuber production in which both marketable and unmarketable tuber yield could be increased [[6], [49], [50]].

And also lower unmarketable tuber yield (2.63 t ha^−1^) was obtained at 40 cm intra-row spacing, whereas higher unmarketable tuber yield (3.47 t ha^−1^) was recorded at 20 cm intra-row spacing (Table 3).This could be due to the presence of intense inter-plant competition at closer spacing and the consequent result of much small sized tubers contribute to the higher unmarketable yield. This result is in agreement with the findings of [33], who stated that the intra-row spacing has a marked effect on unmarketable tuber yield, and the highest unmarketable yield recorded from the closer spacing due to higher inter-plant competition, associated with the small sized tubers.

### 3.9 Shoot fresh weight

Shoot fresh weight of potato crop was significantly influenced (*P* < 0.05) by the main effect of NPS blended fertilizer and intra-row spacing.

The increased application rate of 250 kg NPS ha^−1^ has resulted the highest shoot fresh weight of 385.38 g per plant. While the lowest shoot fresh weight of 261.13 g per plant was recorded in the control treatment (Table 3). The linear increment in the shoot fresh weight of potato crop due to the increased application of blended fertilizer might be because to the role N, P and S in increased rates of blended fertilizers and which has better contribution on biomass accumulation and energy transfer in the crop. The result of present study was also in agreement with the finding of [11,36], who reported that the highest fresh above ground biomass was obtained in the increased fertilizer application.

Similarly, widening the intra-row spacing from 20 cm to 40 cm enhanced the above ground weight yield of a crop. And the highest shoot fresh weight 386.62 g plant^−1^ was obtained in the treatment that was planted at wider intra-row spacing of (40 cm) and the lowest fresh weight 331.80 g per plant was obtained in wider intra-row spacing of 20 cm (Table 3).

The increment in fresh mass of a crop in the wider intra-row spacing might be due to the presence of lesser plant population and which could allow effective utilization of resources such as light, water and nutrients than the crop planted at closer intra-row spacing [[51], [52]].

### 3.10 Shoot dry weight

Shoot dry weight of potato crop was significantly influenced (*P* < 0.05) by the main effect of NPS blended fertilizer and intra-row spacing.

The shoot dry weight has revealed linear increment with an increased rate application of NPS fertilizer. And, the highest shoot dry weight (100.97 g per plant) was obtained in the treatment of rate of 250 kg NPS ha^−1^. Whereas the lowest shoot fresh weight (68.41 g plant^−1^) was obtained in the control treatment (Table 3). The increment in the shoot dry weight of potato crop at a rate of 250 kg NPS ha^−1^ might be due to the an increased role of Nitrogen, Phosphorous and Sulfur nutrients in the blended fertilizer and can contribute wider part in biomass accumulation and also tuber enlargement and production. In line with the findings of this study, above ground dry weight yields of potato plant was significantly influenced by increased application of inorganic fertilization as compared to the control treatment [3].

And also, widening the intra-row spacing from 20 cm to 40 cm has revealed linear increment in the above ground dry weight yield of potato crop. And the highest shoot dry weight (101.27) g plant^−1^ was recorded in the treatment of wider intra-row spacing of (40 cm) and the lowest dry weight (86.93) g plant^−1^ was obtained from in closer intra-row spacing of 20 cm (Table 3). The highest shoot dry weight in the wider intra-row spacing and this might be due to the tuber enlargement with high availability of resources such as light, water and nutrients than the crop planted at closer intra-row spacing.

According to the finding of [3], potato plant produced increased dry mass yield in response to the increased plant spacing.

### 3.11 Harvest index

Harvest index of potato crop was significantly influenced (*P* < 0.05) by the main effect of different rates of NPS fertilizer and intra-row spacing. Increasing the application rate of fertilizer from 0 to 250 kg NPS ha^−1^ increased the harvest index of potato crop from 0.59 to 0.74. The highest harvest index 0.74 was obtained from the treatment that contained fertilizer rate of 250 kg NPS ha^−1^ blended fertilizer which is at par with 200 kg NPS rate application while the lowest harvest index of (0.59) was obtained in the treatment with no fertilizer application (Table 3). Since, harvest index is a measure of the proportion of assimilates partitioned to harvested organs [[6], [51], [53]].

And also, widening the intra-row spacing from 20 cm to 40 cm has increased the harvest index of potato crop from 0.67 to 0.71 Thus, the highest harvest index 0.71 was obtained from the wider plant spacing 40 cm while, the lowest harvest index was from closer intra-row spacing 20 cm. This, the larger harvest index at wider plant spacing might be due to the wider availability for resources like water, nutrient and sun light than that of closer spacing and which can allow the presence of larger assimilates in the tuber portion of the crop [48,43].

The current finding is in line with the findings of [48], who reported that the highest harvest index of potato was recorded from the plots which are planted on the wider intra-row spacing.

Marketable tuber yield of potato crop was significantly influenced (*P* < 0.05) by the interaction effect of NPS blended fertilizer and intra-row spacing. The highest marketable tuber yields 34.29 tone ha^−1^ was recorded at fertilizer application of 250 kg NPS ha^−1^ in combination with intra-row spacing of 20 cm. But the lower marketable tuber yield 28.17 tone ha^−1^ was recorded at the same fertilizer rate of 250 kg NPS ha^−1^ in combination of 40 cm intra-row spacing. The higher marketable tuber yield recorded at a closer spacing combined with the increased application NPS fertilizer which is attributed to more tubers produced at the higher plant population per hectare and the role of NPS fertilizer on tuber formation than that planted in wider intra-row spacing of (40 cm) at the same rate of NPS fertilizer of 250 kg NPS ha^−1^ (Table 4).

**Table 4:** Marketable tuber yield and total tuber yield of potato as influenced by the interaction effect of NPS blended fertilizer and intra-row spacing at Gombora conditions at Hadiya Zone, 2019 cropping season.

Marketable tuber yield (t ha^−1^)						Total tuber yield (t ha^−1^)				
	NPS fertilizer rates (kg ha^−1^)					NPS fertilizer rates (kg ha^−1^)				
Spacing (cm)	0	100	150	200	250	0	100	150	200	250
20	14.68^g^	15.81^f^	21.07^e^	28.17^c^	34.29^a^	18.36^ef^	19.36^e^	24.96^d^	31.70^b^	38.36^a^
30	13.065^gh^	15.34^f^	19.63^e^	26.68^cd^	31.87^b^	16.46^fg^	18.56^e^	23.23^e^	29.93^bc^	36.66^a^
40	12.07^h^	14.92^fg^	17.33^f^	23.92^d^	25.25^d^	15.23^g^	17.90^f^	20.70^e^	26.93^d^	28.80^cd^
LSD (0.5%)		2.41				2.52				
CV (%)		9.55				15.40				
Mean		24.48				24.43				

*Means followed by the same letter are not significantly different at P < 0.05 level of significance, CV = coefficient of variation, NPS = blended Nitrogen, Phosphorous and Sulfur fertilizer and LSD = Least significance difference.

The marketable tuber yield was increased by 57.20% when planted at a rate of 250 kg NPS ha^−1^ with 20 cm intra-row spacing as compared to zero NPS on the same 20 cm intra-row spacing. The present result is also agreed with the findings of many authors [6,54], that they reported increased application of inorganic fertilizer and plant grown in the closer spacing has revealed the higher increment in the marketable yield of a crop.

### 3.12 Total tuber yield (tone ha^−1^)

Interaction effect of blended fertilizer and intra-row spacing has significant influence (*P* < 0.05) on the total tuber yield of potato crop. The highest tuber yield (38.36 t ha^−1^) was obtained from fertilizer application of 250 kg NPS ha^−1^ with the closer intra row spacing of 20 cm, while the lower total tuber yield 28.80 ton per hectare was obtained from the at the same rate of (250 kg NPS ha^−1^) in a wider intra-row spacing of 40 cm (Table 4). However, the lowest amount of total tuber yield (15.23 t ha^−1^) was obtained from the plot which is planted with no fertilizer application at the wider intra-row spacing of 40 cm. It is known that crops grown with in closer plant spacing can have higher availability to get more plant population that plant grown in the wider spacing and this could have direct effect on the total yield.

This is supposed to be due to the compensation effect of closer intra row spaced plants per hectare with the higher rates of NPS fertilizer, which resulted in higher yield of tubers per plant. This result is in agreement with the finding of [5,19,23], who reported that tuber yield per hectare was reduced due to the shortage of mineral nutrients and insufficient number of plants grown per hectare in wider intra-row spacing as compared to the plants grown at closer intra row spacing [[55], [56], [57]].

Total fresh weight (g plant^−1^).

Total fresh weight of potato was significantly influenced (*P* < 0.05) by the interaction effect of NPS blended fertilizer and intra-row spacing.

The result of this study shown that increasing application rate of NPS blended fertilizer from 0 to 250 kg ha^−1^ and reducing the intra-row spacing from 40 cm to 20 cm has linear increment on the total fresh weight yield of a crop. That is, the highest total fresh mass yield 1274.00 g plant^−1^ was obtained in the combined effect of higher NPS rate fertilizer of 250 kg NPS ha^−1^ in narrower intra-row spacing of 20 cm and the lower 1165.2 g plant^−1^ was recorded with the fertilizer application rate of 250 kg NPS ha^−1^ in the same spacing (20 cm) while, the lowest total fresh weight yield 756.60 g plant^−1^ was obtained in the treatment that planted without the application of NPS fertilizer in the wider intra-row spacing (40 cm) (Table 5). This might be due the role of N, P and S fertilizers and that P is known as a part of structure of DNA, RNA, ATP, phospholipids in the membranes and also Nitrogen and Sulfur fertilizer can also plays an important role in carbohydrate metabolism and energy transfer systems of a crop. This can help the crop to accumulate an increased biomass yield with the increased NPS fertilization application by narrowing intra-row spacing.

**Table 5:** Total fresh mass of potato crop as influenced by the interaction effect of interaction effect of NPS blended fertilizer and intra-row spacing at Gombora Woreda condition in Hadiya Zone, 2019 summer cropping season.

Total fresh mass (g plant^−1^)					
	NPS fertilizer rates (kg ha^−1^)				
Spacing (cm)	0	100	150	200	250
20	756.60^f^	982.20^de^	1161.10^bc^	1115.31^c^	1165.20^b^
30	802.52^ef^	1016.62^d^	1115.90^c^	1212.71^a^	1224.22^a^
40	853.20^e^	1108.31^cd^	1197.88^b^	1244.60^a^	1274.2^a^
LSD (0.5%)	61.20				
CV (%)	7.50				
Mean	1082.04				

Whereas, means followed by the same letter(s) in rows and columns under each parameter are not significantly different at *P* < 0.05 level of significance, NPS= Nitrogen, phosphorous and sulfur blended fertilizer. LSD = Least significance difference, CV = coefficient of variation.

Similarly [8], reported that increased fertilizer application revealed the highest biomass yield and [44,58,59] reported that narrower plant spacing has shown increment in total biomass yield of potato crop. Therefore, the increased total fresh biomass yield could be due to the combined effect of increased NPS fertilizer and high plant population that could exist per unit area at narrow (20 cm) intra-row spacing.

Underground fresh and dry weight (g plant^−1^).

Underground fresh and dry weight of potato crop had significantly influenced (*P* < 0.05) by the interaction effect of NPS blended fertilizer and intra-row spacing. In this experiment, fresh weight of underground part of the crop was increased with the application of NPS fertilizer in combination with closing intra-row spacing. Maximum underground fresh mass 869.87 g plant^−1^ was obtained at blended NPS fertilizer rate of 250 kg NPS ha^−1^ in closer intra-row spacing of 20 cm, whereas, the reduced underground fresh mass 835.24 g plant^−1^ was recorded at a blended NPS fertilizer rate of 250 kg NPS ha^−1^ with wider intra-row spacing of 40 cm (Table 6). However, minimum underground fresh mass of 531.40 g plant^−1^ was recorded in the treatment that planted in closer spacing with no NPS application. According the findings of [[34], [56], [57]] higher plant population per unit area at closer intra-row spacing has enhanced efficient resources utilization with an increased application of inorganic fertilizer and increase in biomass.

**Table 6:** The interaction effect of blended NPS fertilizer rates and intra-row spacing has significant influence on underground fresh and dry mass of potato crop at Gombora condition, Hadiya Zone during 2019 summer cropping season.

NPS fertilizer rates (kg ha^−1^)						NPS fertilizer rates (kg ha^−1^)				
	Underground fresh mass (g plant^−1^)					Underground dry mass (g plant^−1^)				
Spacing (cm)	0	100	150	200	250	0	100	150	200	250
20	531.40^d^	676.67^cde^	722.72^c^	844.64^a^	869.87^a^	114.80^h^	155.85^e^	196.55^c^	206.58^b^	212.88^a^
30	548.80^d^	676.83^c^	807.82^b^	840.98^a^	863.51^a^	123.92^g^	159.82^e^	194.15^c^	205.62^b^	206.15^b^
40	566.03^d^	682.84^c^	810.60^b^	855.56^a^	835.24^ab^	139.02^f^	171.32^d^	196.55^c^	208.42^ab^	204.72^b^
LSD (0.05)		56.70				6.52				
CV (%)		5.84				6.98				
Mean		742.29				179.76				

Whereas, means followed by the same letter (s) in rows and columns under each parameter are not significantly different at *P* < 0.05 level of significance, NPS= Nitrogen, phosphorous and sulfur blended fertilizer, LSD = Least significance difference, CV = coefficient of variation.

And similarly, the dry mass of underground part of potato crop was significantly influenced (*P* < 0.05) by the interaction of increased application of NPS blended fertilizer and different intra-row spacing. The results of present study indicated that underground dry weight of potato crop was increased with increase application of blended NPS fertilizer in combination with the closing intra-row spacing from 40 to 20 cm. Maximum underground dry mass 212.88 g plant^−1^ was obtained when applying blended NPS fertilizer rate of 250 kg NPS ha^−1^ at intra-row spacing of 20 cm, whereas, the reduced underground dry 204.72 g plant^−1^ was obtained at a blended NPS fertilizer rate of 250 kg NPS ha^−1^ under wider intra-row spacing of 40 cm (Table 6). Similarly, raise in dry mass of potato crop might indicate the importance of blended NPS fertilizer application in biomass production, carbohydrate metabolism and energy transfer systems.

Moreover, high plant population per unit area at closer intra-row spacing has enhanced efficient resources utilization whereas low dry weight resulted due to low plant population in wider intra-row spacing could be due to inadequate amount application of blended fertilizer [[44], [58], [60], [61]].

## 4 Discussion

Potato (Solanum tuberosum L.) is the world's fourth most important food crop after maize, rice and wheat. The field experiment was conducted to determine the effect of five rates of blended fertilizer (0, 100, 150, 200, and 250) kg NPS ha^−1^ and intra-row planting spacing of 20 cm, 30 cm and 40 cm and laid out by randomized complete block design with three replication in a factorial arrangement. The analysis of variance revealed that, almost all measured parameters such as marketable tuber yield, total tuber yield, stem number per hill, total fresh mass, underground fresh and dry mass were significantly (*P* < 0.05) influenced by the interaction of Nitrogen, Phosphorous and Sulfur (NPS blended fertilizer) and intra-row spacing.

More specifically, the highest plant height (96.60 cm), highest marketable tuber yield (34.29 tha^-1^), highest total tuber yield (38.36 t ha^−1^) and highest total fresh biomass (1274.2 g plant^−1^) were recorded from NPS rate of 250 kg NPS ha^−1^ and intra-row spacing of 20 cm while the lowest recorded from control treatment in wider intra-row spacing (40 cm). Plants grown at closer intra-row spacing of 20 cm has shown a significant increase at total tuber yield of potato with the combined effect of higher rate of NPS fertilizer; might might be because of the increment total population and the role of inorganic fertilizers.

And also at higher rate of NPS blended fertilizer application, plants might observe sufficiently available resource, wider ability to get them and increased photosynthetic efficiency with the help of those essential nutrients from blended inorganic fertilizer that further increased the vegetative growth and ultimately resulted in increased leaf area per hill, wider leaf area, stem number and tuber number. Therefore, the increased performance at above mentioned parameters at higher rates of NPS fertilizer might be due to increased epidermal cell expansion and cell development with the sufficiency of nutrients.

In consistent to this study [39], confirmed that leaf area per hill increased in the high fertilizer rate and that the wider leaf area per hill was obtained from higher fertilizer rate application. In general potato planted at narrower intra-row spacing of 20 cm in combinations with rate of (250 kg NPS ha^−1^) has resulted in better performance on the growth, plant height, tuber number per hill, tuber weight, marketable yield and harvest index and tuber yield and this might be due to the increment of plant population and the role of N, P and S fertilizers. This revealed that increased application of NPS blended fertilizer at narrower intra-row spacing might be a possible solution for intensive production of potato crop and for its increased yield [.

From this, it is possible to made a partial conclusion thus, the farmers can cultivate by using the closer intra-row spacing of 20 cm in combination the increased rate application of inorganic fertilizer to get better performance of the potato crop [[55], [57]]..

## 5 Conclusion

Over all, in the present study, potato variety responded to a higher rate of NPS blended fertilizer on narrower intra-row spacing by enhancing its growth and yield. However, the plant phenology parameters, such as 50% emergence, 50% flowering and days to 90% physiological maturity were fastened in response to an declining rate of NPS blended fertilizer application and narrowing intra-row spacing from 40 cm to 20 cm. Most of the parameters among phenology yield and yield have shown significant difference due to the main effect of NPS blended fertilizer and intra-row spacing.

However, average stem per hill, marketable tuber yield, total tuber yield, the total fresh biomass yield, underground fresh and dry mass of the crop were significantly influenced with the interaction effect of NPS fertilizer and intra-row spacing. Taken all together, narrower intra-row spacing of 20 cm in combinations with rate of (250 kg NPS ha^−1^) resulted in better performance of the potato variety in terms of growth, harvest index and tuber yield. This revealed that increased application of NPS blended fertilizer at narrower intra-row spacing might be a possible solution for intensive production of potato crop and for its increased yield.

In this study, the highest values of plant height (96.60 cm), marketable tuber yield (34.29 tha^-1^), total tuber yield (38.36 t ha^−1^) and total fresh biomass (1274.2 g plant^−1^) were recorded from the increased rate application of 250 kg NPS ha^−1^ at the closer intra-row spacing of 20 cm while the lowest values at each parameters were recorded from control treatment at wider intra-row spacing (40 cm). Therefore, the application of 250 kg NPS ha^−1^ with an intra-row spacing of 20 cm cm can give an increased tuber yield and then it could be recommended for the production of potato in the study area.

## Author contribution statement

Temesgen Tadesse Mamiru: Conceived and designed the experiments; Performed the experiments; Analyzed and interpreted the data; Contributed reagents, materials, analysis tools or data; Wrote the paper.

Getachew Mulugeta Geleto: Analyzed and interpreted the data; Contributed reagents, materials, analysis tools or data; Wrote the paper.

## Funding statement

This research did not receive any specific grant from funding agencies in the public, commercial, or not-for-profit sectors.

## Data availability statement

The data that has been used is confidential.

## Additional information

No additional information is available for this paper.

## Declaration of competing interest

The authors declare no conflict of interest.
